# 

**Published:** 1934

**Authors:** 


					I. LI. No. 192.
hk ?
SUMMER, 1934.
m.
The Bristol
Journal
E. WATSON-WILLIAMS, M.O., Ch.lL; F.R.C.S., Assistant Editor.
Editorial Secretary : A. L. FLEMMING, M.B., Ch-B.
mm-i
PUBLISHED UNDER THE AUSPICES OF
THE BRISTOL MEDICO-CHIRURGICAL SOCIETY.
Editor : J. A. NIXON, M.D., F.R.C.P.
- C ; : : ? ? 'A. yr .
WITH WHOM ABE ASSOCIATED
E. W. HEY GROVES, M.S., F.R.C.S., D.Sc.
A. E. ILES, O.B.E., M.B., B.S., F.R.C.S.
A. RENDLE SHORT, B.Sc., M.D., B.S., F.R.O.S.
of*5
BRISTOL: J. W. ARROWSMITH LTD., 11 QUAY, STREET.
London : J. W. Abrowsmith (London) Ltd.*, 8 Endsleiqh Gardens, W.G. 1.
? '
Price Three Shillings net.
m
BHUtt " ? ?? ? ? - ^ , P ? V - 3.
v ? 'Hfl
>- K v., -;:.. ?; ? . *-? -

				

